# Metformin prevents peritendinous fibrosis by inhibiting transforming growth factor-β signaling

**DOI:** 10.18632/oncotarget.21695

**Published:** 2017-10-09

**Authors:** Wei Zheng, Jialin Song, Yuanzheng Zhang, Shuai Chen, Hongjiang Ruan, Cunyi Fan

**Affiliations:** ^1^ Department of Orthopaedics, Shanghai Jiao Tong University Affiliated Sixth People's Hospital, Shanghai, China; ^2^ Changhai Hospital of Second Military Medical University, Shanghai, China

**Keywords:** metformin, peritendinous fibrosis, TGF-β, AMPK

## Abstract

Injury-induced peritendinous adhesion is a critical clinical problem that leads to tendon function impairment. Therefore, it is very urgent to explore potential approaches to attenuate peritendinous adhesion formation. Recently, several studies have demonstrated the biological effect of metformin in inhibiting multiple tissue fibrosis. In this study, we performed *in vitro* and *in vivo* experiments to examine whether metformin prevents injury-induced peritendinous fibrosis. We found that tendon injury induced severe fibrosis formation in rats. However, orally administered metformin significantly alleviated the fibrosis based on macroscopic and histological evaluation. Peritendinous tissue from metformin-treated rats also showed decreased expression of fibrotic genes including col1a1, col3a1, and α-smooth muscle actin (α-SMA), and inhibition of transforming growth factor (TGF)-β1 signaling. The cell counting kit (CCK)-8, flow cytometry, and 5-ethynyl-2′-deoxyuridine (EdU) staining analyses showed that treatment of NIH/3T3 fibroblasts with metformin significantly inhibited excessive cell proliferation and promoted cell apoptosis. Metformin treatment also inhibited the expression of fibrotic genes and decreased the phosphorylation of smad2/3 and extracellular signal-regulated kinase (ERK) 1/2. Furthermore, blocking AMP-activated protein kinase (AMPK) signaling abolished the inhibitory effect of metformin on fibrosis. Our findings indicate that metformin has a protective role against peritendinous tissue fibrosis and suggest its clinical use could be a promising therapeutic approach.

## INTRODUCTION

Peritendinous tissue adhesion after tendon injury is an important orthopedic condition that causes gliding dysfunction as well as pain and requires complex surgical intervention [1]. Presently, multiple strategies including drugs, physical barriers, and physiotherapy are used to prevent peritendinous tissue adhesion [2–5]. Researchers have explored various pharmaceutical approaches, especially nonsteroidal anti-inflammatory drugs (NSAIDs), to prevent adhesion formation [6]. However, the efficiency of these drugs is suboptimal and can lead to increased side effects.

A key mediator in the pathogenesis of tendon adhesion is transforming growth factor (TGF)-β1, which is an important factor in the pathogenesis of tendon adhesion [7, 8]. TGF-β1 promotes fibroblast to myofibroblast differentiation (FMD) and myofibroblast proliferation by mediating various signaling pathways, including the canonical TGF-β/Smad2/3 and noncanonical mitogen-activated protein kinase (MAPK) pathways [9].

In the past decades, metformin has become one of the most extensively used oral anti-hyperglycemic medicines for patients with type 2 diabetes. Recent studies have revealed additional biological functions of metformin including a potential protective role in preventing tissue fibrosis in the kidney, lung, heart, and liver [9–12]. However, whether metformin attenuates injury-induced peritendinous adhesion remains unknown.

In the present study, we sought to answer the following questions: (1) whether metformin inhibits peritendinous tissue fibrosis induced by injury and (2) whether metformin inhibits TGF-β1 signaling activation.

## RESULTS

### Metformin alleviates injury-induced tendon adhesion *in vivo*

**Figure 1:**
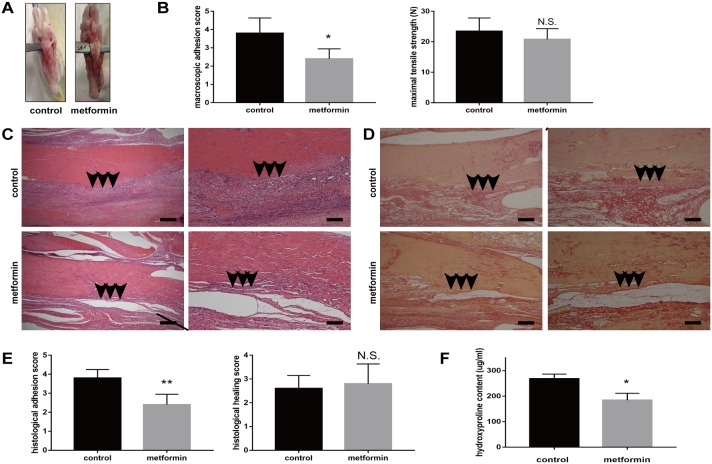
Metformin treatment reduces peritendinous tissue adhesion in tendon-injured rats **(A)** Macroscopic images showed less adhesive tissues in metformin-treated (200 mg/kg/d) rats. **(B)** Gross adhesion scores (left) were significantly lower in metformin-treated rats compared with control rats 3 weeks after tendon operation (2.8 vs 3.9, p = 0.005). Maximal tensile strengths (right) were comparable between control and metformin-treated rats (23 vs 21 N, p = 0.173) (n = 8 for each group). **(C)** HE staining of peritendinous tissues showed reduced cell proliferation and inflammation by metformin treatment. Arrows indicate adhesion formation. Scale bars (left) = 250 μm. Scale bars (right) = 100 μm. **(D)** Sirius-red staining revealed decreased collagen deposition by metformin treatment. Arrows indicate excessive collagen deposition. Scale bars (left) = 250 μm. Scale bars (right) = 100 μm. **(E)** Histological scores for adhesion decreased after metformin treatment (2.4 vs 3.8, p = 0.002). Histological healing scores were comparable between control and metformin-treated rats (2.6 vs 2.8, p = 0.667) (n =5 for each group). **(F)** Metformin treatment significantly deceased the hydroxyproline (Hyp) content of tendon tissues at 3 weeks (184 vs 268 ug/ml, p = 0.011) (n = 5 for each group). Data are expressed as means ± SEM. ^*^ P < 0.05; ^**^ P < 0.01; N.S. not significant.

We first established a peritendinous adhesion rat model by surgery. Three weeks later, all the rats were euthanized and tendons were examined. All the rats survived the experimental period. The gross observation showed thick adhesion tissue around the tendons in the control group, whereas this effect significantly decreased in the metformin-treated group (Figure 1A). The gross adhesion grading scores of the peritendinous tissues were significantly lower in metformin-treated group (p = 0.005, Figure 1B). No significant difference in maximal tensile strength was observed between the control and metformin-treated groups (p = 0.173, Figure 1B).

Histological evaluation using hematoxylin and eosin (H&E) staining showed that rats in the control group exhibited more extensive proliferation of fibroblasts and infiltration of inflammatory cells than the metformin-treated rats did (Figure 1C). Sirius-red staining showed massive collagen deposition after tendon injury (Figure 1D). The Sirius-red staining revealed that oral administration of metformin reduced collagen deposition (Figure 1D). Histological adhesion scoring of tendons also showed more extensive adhesion in the control group (p = 0.002, Figure 1E). Histological tendon healing scores showed no significant difference between control and metformin-treated rats (p = 0.667, Figure 1E). We further measured the hydroxyproline (Hyp) content, which is a major constituent of collagen, to quantify the extent of adhesion. Rats in the control group showed significantly higher levels of Hyp than the metformin-treated rats did (268 and 184 μg/mL, respectively, p = 0.011, Figure 1F). Taken together, our data suggest a protective role for metformin in alleviating injury-induced exacerbation of peritendinous adhesion *in vivo*.

### Metformin inhibits proliferation and TGF-β1 signaling pathway *in vivo*

**Figure 2:**
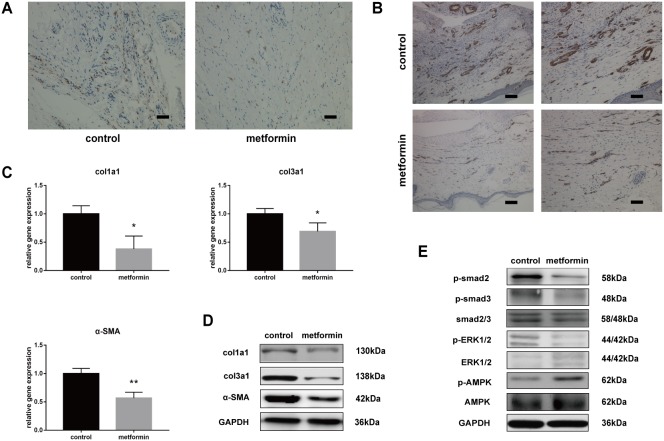
Metformin inhibits cell proliferation and TGF-β1 signaling pathway *in vivo* **(A)** Ki67 assay showed that metformin inhibited abnormal cell proliferation (brown) in peritendinous tissues. Scale bar = 50 μm. **(B)** Immunohistochemistry (IHC) for α-SMA (brown) (b) showed significantly reduced positive cells in metformin-treated rats. Scale bar (left) = 100 μm. Scale bar (right) = 50 μm. The real-time PCR **(C)** and western blot **(D)** analysis showed that both mRNA and protein levels of col1a1, col3a1 and α-SMA in peritendinous tissues decreased after metformin treatment. **(E)** The western blot analysis showed that metformin treatment significantly inhibited canonical (SMAD) and noncanonical (MAPK) TGF-β signaling pathways. Data are expressed as means ± SEM. ^*^ P < 0.05; ^**^ P < 0.01.

Excessive cell proliferation, persistent myofibroblast activation, and extensive extracellular matrix (ECM) protein synthesis are vital for tendon adhesion formation. Therefore, we demonstrated whether metformin could inhibit proliferation and the TGF-β1 signaling pathway *in vivo*. The Ki67 staining revealed that metformin treatment significantly decreased excessive cell proliferation (Figure 2A).

Smooth muscle actin (SMA) is a marker of myofibroblasts, and activation of myofibroblasts has been shown to promote fibrogenesis. Immunohistochemistry (IHC) for SMA showed intensive positive staining in tendon tissue sections of injured rats, whereas the number of positively stained cells was reduced after metformin treatment (Figure 2B). To evaluate the effect of metformin on fibrotic gene expression, we performed reverse transcription-polymerase chain reaction (RT-PCR) and western blotting in tendon tissues.

The mRNA expression of col1a1, col3a1, and SMA decreased in metformin-treated rats compared with that in the controls (Figure 2C). Western blotting also showed significantly reduced protein levels of col1a1, col3a1, and SMA in the treatment group (Figure 2D). Furthermore, we evaluated the effect of metformin on the TGF-β1 signaling pathway. In the metformin-treated group, the protein level of phosphorylated-AMP-activated protein kinase (p-AMPK) was significantly elevated. Rats treated with metformin showed significantly decreased phosphorylation levels of smad2/3 and extracellular signal-regulated kinase (ERK)1/2, which indicates that metformin inhibited the SMAD and MAPK pathways in injured peritendinous tissues (Figure 2E).

### Metformin induces progressive apoptosis and inhibits proliferation *in vitro*

**Figure 3:**
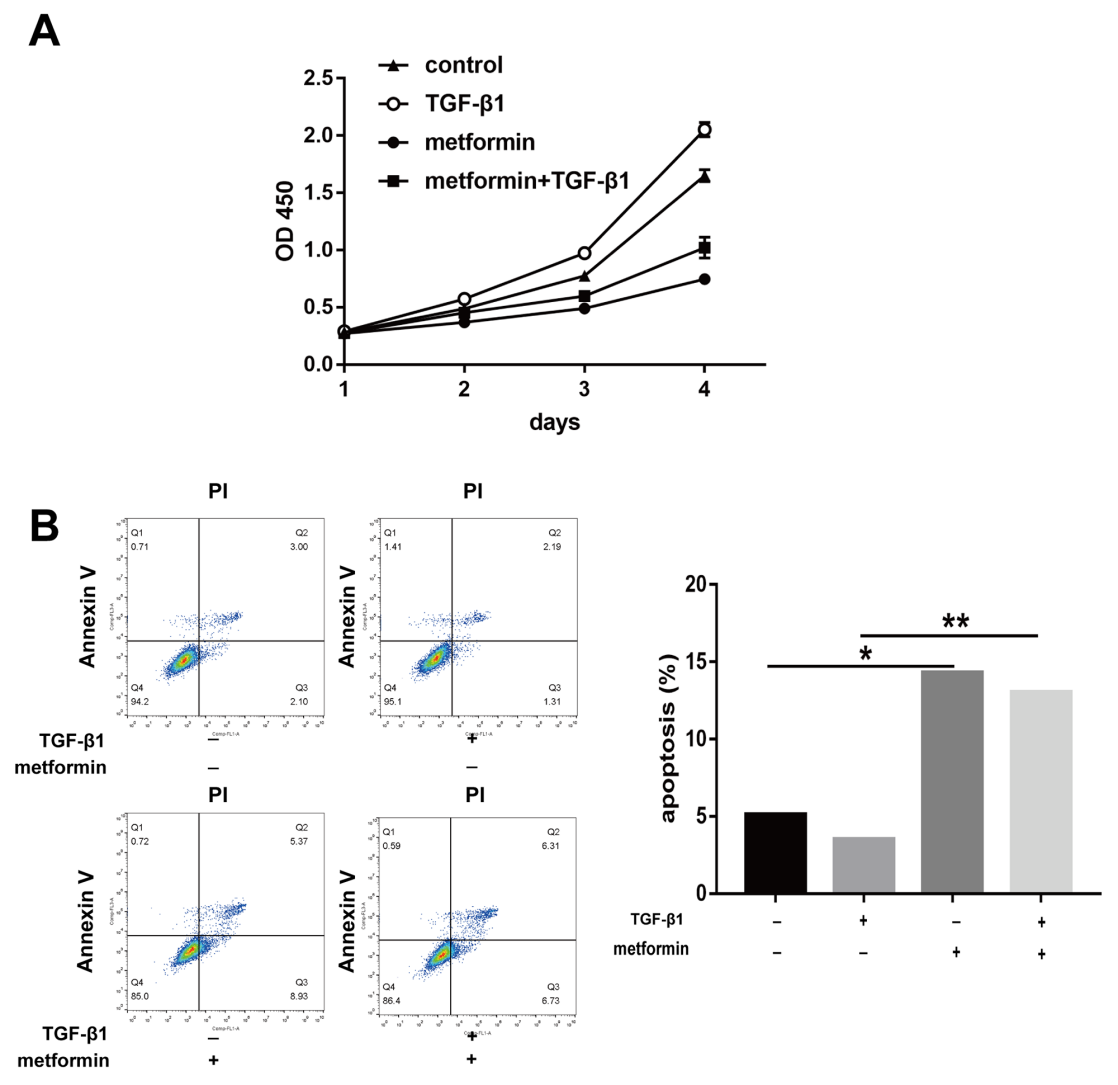
Metformin induces progressive apoptosis *in vitro* **(A)** NIH/3T3 fibroblasts were treated for 24h, 48h, 72h, and 96h, respectively. Cell Counting Kit-8 (cck8) assay showed that cell viability was repressed by metformin treatment. **(B)** Metformin induced a significantly increased apoptosis assessed by flow cytometry in fibroblasts. The histogram showed the percentage of apoptotic cells. Data are expressed as means ± SEM. ^*^ P < 0.05; ^**^ P < 0.01.

Excessive fibroblast proliferation is a vital pathological characteristic of peritendinous tissue fibrosis. The cell counting kit (CCK)-8 showed that metformin decreased the TGF-β1-induced fibroblast viability (Figure 3A). Flow cytometric analysis of cell apoptosis showed that TGF-β1 decreased fibroblast apoptosis, while metformin alone or in combination with TGF-β1 promoted apoptosis in NIH/3T3 fibroblasts (Figure 3B).

**Figure 4:**
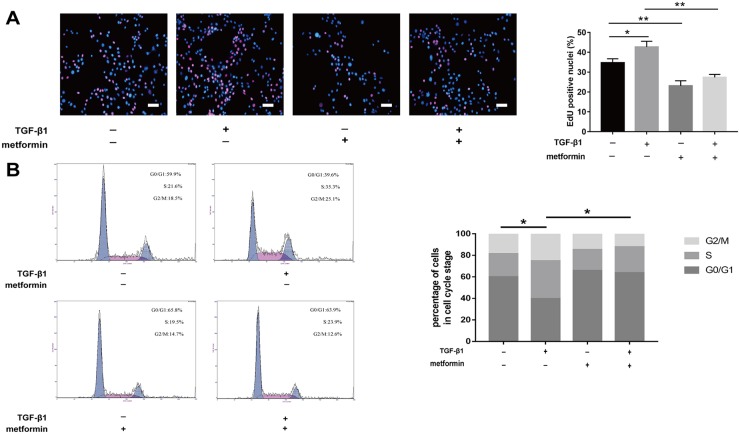
Metformin inhibited fibroblast proliferation *in vitro* **(A)** EdU staining was used to detect proliferative fibroblasts (red). Metformin markedly reduced the percentage of proliferative cells. **(B)** fibroblasts were treated as indicated for 48h and analyzed by flow cytometry to estimate the distribution of fibroblasts in each phase of the cell cycle. TGF-β1 treatment significantly increased S and G2/M phase cells, whereas metformin attenuated the transition. The histogram represents the percentage of cells in each cell cycle phase. Data are expressed as means ± SEM. ^*^ P < 0.05; ^**^ P < 0.01.

The 5-ethynyl-2′-deoxyuridine (EdU) staining also revealed that TGF-β1 induced fibroblast proliferation, while cotreatment with metformin inhibited this effect (Figure 4A). As shown by the cell cycle analysis, the number of G0/G1 NIH/3T3 fibroblasts increased after metformin treatment (Figure 4B). The number of S and G2/M phase cells increased after TGF-β1 treatment while the effect was abolished by treatment with metformin. Taken together, our data showed that metformin inhibited excessive cell proliferation and promoted apoptosis of NIH/3T3 cells.

### Metformin decreases TGF-β1-induced adhesion *in vitro*

**Figure 5:**
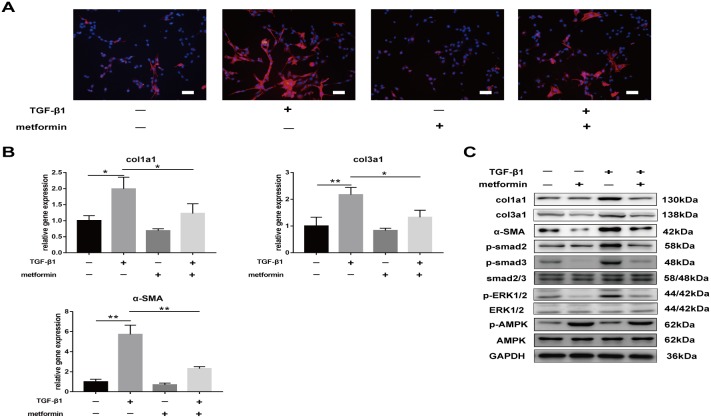
Metformin decreases TGF-β1-induced adhesion *in vitro* **(A)** Immunofluorescent (IF) staining showed that the expression of α-SMA positive (red) fibroblasts was inhibited by metformin treatment for 24 h. scale bar = 50 μm. **(B)** The real-time PCR analysis showed that mRNA expression levels of col1a1, col3a1 and α-SMA in NIH/3T3 fibroblasts were inhibited by metformin treatment. **(C)** The western blot analysis showed that protein levels of fibrotic genes were inhibited by metformin treatment. Metformin treatment increased phosphorylation of AMPK, and inhibited both SMAD and MAPK signaling pathways in NIH/3T3 fibroblasts. Data are expressed as means ± SEM. ^*^ P < 0.05; ^**^ P < 0.01.

We sought to determine whether metformin decreased TGF-β1-induced peritendinous fibrosis *in vitro*. Immunofluorescence (IF) staining of NIH/3T3 fibroblasts showed that treatment with 2 ng/mL TGF-β for 24 h increased the expression of SMA. However, treatment with 5 mM metformin decreased the expression of SMA and inhibited the increased expression of SMA induced by TGF-β1 (Figure 5A).

Real-time PCR and western blotting further confirmed that metformin decreased TGF-β1-induced expression of fibrosis-related genes, including col1a1, col3a1, and SMA (Figure 5B and 5C). Furthermore, TGF-β1 activated canonical and noncanonical signaling pathways, including p-smad2/3 and p-ERK1/2. Metformin treatment decreased the expression of phosphorylated proteins activated by TGF-β1 (Figure 5C). These results suggest that metformin inhibited TGF-β1-induced peritendinous tissue fibrosis *in vitro*.

### Metformin decreases TGF-β1-induced fibrosis by AMPK activation-dependent mechanism

**Figure 6:**
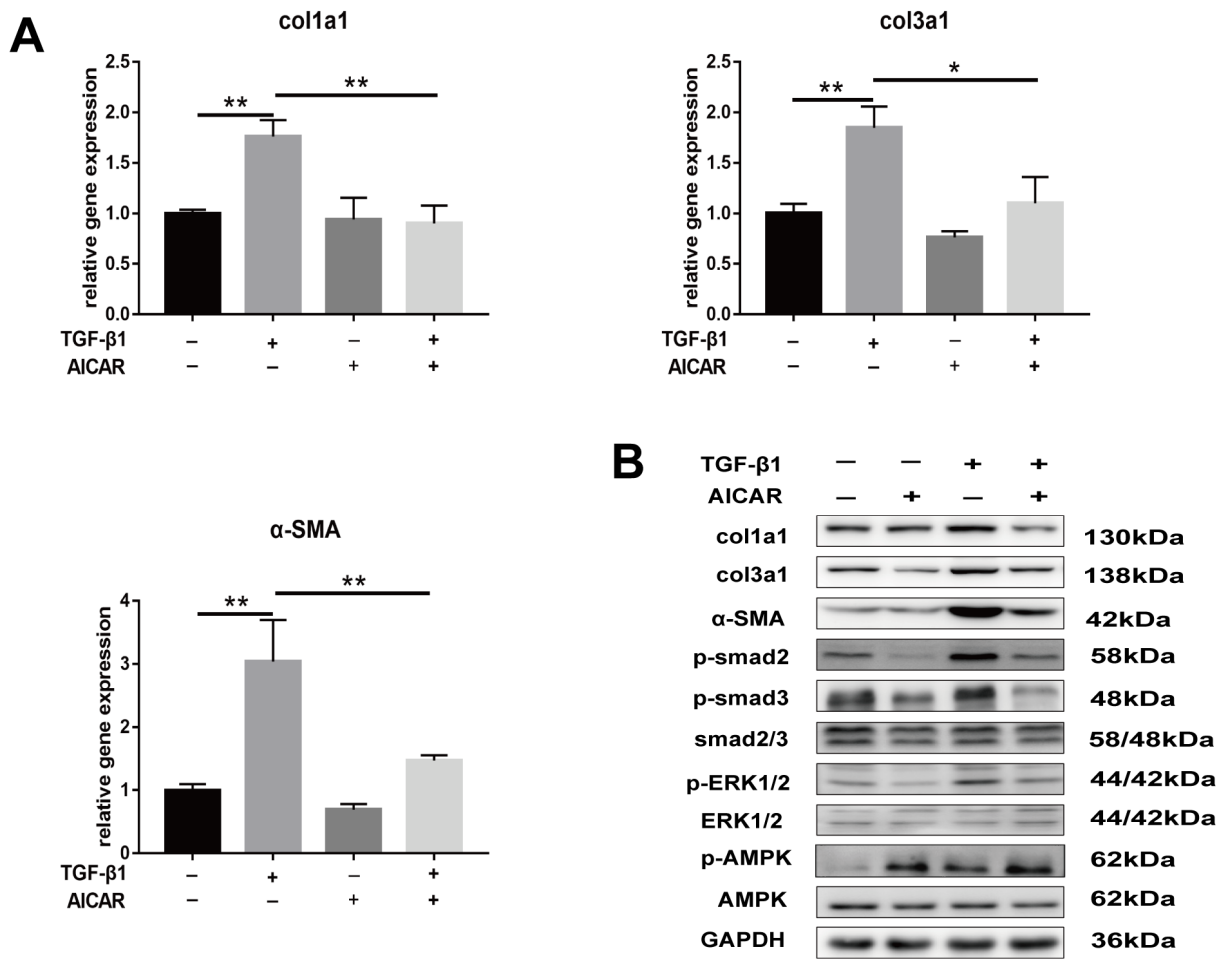
Activation of AMPK signaling pathway inhibited TGF-β1 induced fibrosis in fibroblasts **(A)** AICAR (1 mM) markedly decreased the mRNA levels of col1a1, col3a1 and α-SMA induced by TGF-β1 in NIH/3T3 fibroblasts. **(B)** AICAR markedly decreased the protein levels of col1a1, col3a1 and α-SMA induced by TGF-β1 in NIH/3T3 fibroblasts. Both the SMAD and MAPK signaling pathways were inhibited by AICAR treatment in NIH/3T3 fibroblasts. Data are expressed as means ± SEM. ^*^ P < 0.05; ^**^ P < 0.01.

Our results showed that metformin activated the phosphorylation of AMPK *in vitro* and *in vivo*. Furthermore, we demonstrated that metformin inhibited TGF-β1-induced peritendinous tissue fibrosis through AMPK activation. First, we evaluated whether activation of AMPK signaling pathway inhibited peritendinous fibrosis formation. Real-time PCR showed that fibroblasts treated with 1mM 5-aminoimidazole-4-carboxamide1-β-D-ribofuranoside (AICAR), a well-known AMPK activator, exhibited a significant downregulation of mRNA and protein levels of fibrotic genes (Figure 6A and 6B). Then we inhibited AMPK by pretreated with compound C before TGF-β1 or metformin treatment or both.

**Figure 7:**
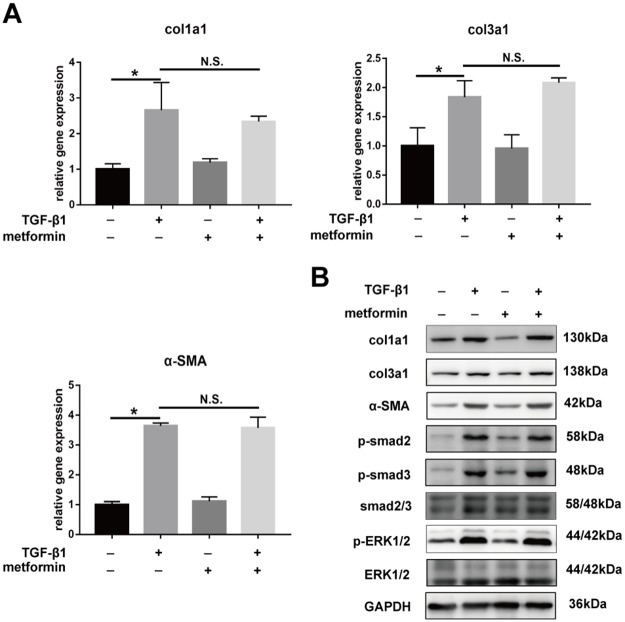
Inhibitory effect of metformin on peritendinous fibrosis was blocked by Compound C All the NIH/3T3 fibroblasts were pretreated with Compound C (1 μM) for 24 h before further treated with TGF-β1 (2 ng/ml) and/or metformin (5 mM). **(A)** The real-time PCR analysis showed that metformin was unbale to down-regulated the mRNA levels of col1a1, col3a1 and α-SMA in fibroblasts pretreated with Compound C. **(B)** The western blot analysis showed that metformin was unbale to down-regulated the protein levels of col1a1, col3a1 and α-SMA in fibroblasts pretreated with Compound C. Compound C abolished the down-regulation of metformin on SMAD and MAPK signaling pathways. Data are expressed as means ± SEM. ^*^ P < 0.05; ^**^ P < 0.01. N.S. not significant.

**Figure 8:**
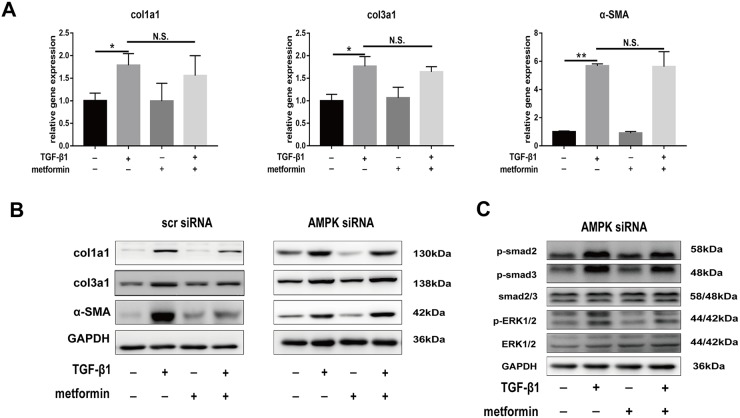
Small interfering-RNA (siRNA) targeting AMPK blocked inhibitory effect of metformin on decreasing expression of fibrotic genes and TGF signaling pathway All the NIH/3T3 fibroblasts were pre-transfected with scramble siRNA or AMPK siRNA. **(A)** Metformin was unable to down-regulated the mRNA levels of col1a1, col3a1 and α-SMA in fibroblasts pre-transfected with AMPK siRNA. **(B)** Metformin significantly reduced the protein levels of col1a1, col3a1 and α-SMA in fibroblasts pre-transfected with scramble siRNA. However, the inhibitory effect was abolished by AMPK siRNA. **(C)** In fibroblasts pretreated with AMPK siRNA, metformin was unable to inhibited SMAD or MAPK signaling pathways. Data are expressed as means ± SEM. ^*^ P < 0.05; ^**^ P < 0.01. N.S. not significant.

The real-time PCR and western blotting showed that compound C abolished the metformin-induced downregulation of col1a1, col3a1, and SMA (Figure 7A). Further, we transfected fibroblasts with small interfering RNA (siRNA) targeting AMPK or negative control. The results showed that pre-transfection of fibroblast with AMPK siRNA blocked the inhibitory effect of metformin on fibrosis (Figure 8A and 8B). Furthermore, the metformin-induced inhibition of the activation of canonical (SMAD) and noncanonical (MAPK) TGF-β1 signaling pathways was abolished by compound C (Figure 7B) or AMPK siRNA (Figure 8C). These *in vitro* studies suggested that metformin might inhibit TGF-β1-induced peritendinous tissue fibrosis by activating AMPK signaling.

## DISCUSSION

Peritendinous tissue adhesion, characterized by excessive peritendinous ECM deposition and subsequent tendon dysfunction, is a major clinical complication of tendon injury. Multiple strategies to address this condition, including physical anti-adhesion barriers and drugs, have been extensively studied [2–4, 13]. However, the effect of these approaches remains uncertain and, therefore, an effective measure to inhibit tendon adhesion at key locations is required.

Although metformin has been used as an oral antidiabetic drug for decades, recent studies have uncovered its novel biological effects including antitumor and antifibrotic activities [9, 10, 12, 14–16]. In the present study, we demonstrated that metformin alleviated peritendinous tissue fibrosis lesions after tendon injury. First, we showed that metformin inhibited abnormally activated proliferation *in vitro* and *in vivo*. Activation of TGF-β1 signaling has previously been shown to promote fibrosis in multiple tissues [17, 18]. *in vitro* studies have also demonstrated that TGF-β1 strongly promoted cell proliferation and inhibited apoptosis [9, 19]. Our previous studies have reported the activation of proliferation in TGF-β1-stimulated fibroblasts and adhesive tendon tissue [20, 21]. However, TGF-β1-treated NIH/3T3 fibroblasts exposed to metformin showed decreased cell viability, and metformin treatment both promoted fibroblast apoptosis and inhibited proliferation. Li showed that TGF-β1 treatment resulted in reduced apoptosis and increased proliferation of HFL-1 cells, whereas metformin cotreatment abolished these effects [9]. It has been established that the cell cycle is inhibited mainly by G0/G1 arrest [14, 22, 23]. Our results also showed that metformin promoted G0/G1 arrest of TGF-β1-treated fibroblasts.

Second, we showed that metformin exhibited a potent antifibrotic effect by inhibiting the expression of fibrotic markers such as col1a1 and col3a1. Recent studies have shown that myofibroblasts, the activated form of fibroblasts, are the major source of excessive ECM deposition in multiple tissue fibrosis [17, 24]. In animal models of aldosterone and salt-induced cardiac fibrosis, metformin treatment significantly reduced the expression of fibrotic genes in the heart [11]. We found that metformin inhibited the increased expression of α-SMA in activated fibroblasts. Cavaglieri recently reported that metformin significantly reduced macrophage infiltration, expression of inflammation markers, and deposition of ECM, thereby reducing interstitial fibrotic lesions in a mouse with unilateral ureteral obstruction [15].

Finally, our study revealed that metformin inhibited TGF-β1-induced peritendinous adhesion by activating the AMPK signaling pathway. Smad2/3 and ERK1/2 have previously been reported to be important downstream mediators of TGF-β1 signaling [7, 25]. The ERK1/2 pathway was initially considered a growth-promoting pathway, mediating the transition from the G1 to the S phase and DNA synthesis during the S phase [26]. A previous study has also shown that activation of ERK1/2 and smad2/3 mediated collagen synthesis [25]. Li found that oral metformin treatment inhibited the phosphorylation of both smad2/3 and ERK1/2, and attenuated gefitinib-induced pulmonary fibrosis [9]. Studies have also shown that metformin exerted a protective effect against tumor progression by inhibiting TGF-β1 signaling [27, 28].

We have demonstrated that persistent activation of canonical and noncanonical TGF-β1 signaling promoted peritendinous tissue adhesion formation in tendon-injured animal models. Our previous study showed that the continuous local release of celecoxib significantly inhibited the activation of smad2/3 and ERK1/2, which prevented tendon adhesion [6]. Nevertheless, local celecoxib treatment was shown to slightly impair tendon healing [6]. The results of the present study showed that metformin effectively inhibited the *in vitro* and *in vivo* phosphorylation of smad2/3 and ERK1/2. In addition, it is noteworthy that the indexes for tendon healing show no significantly difference between control and metformin-treated rats.

The AMPK pathway is the best known metformin-mediated pathway. Metformin reduces the ATP/AMP ratio by suppressing mitochondrial respiratory-chain complex I. Excessive AMP binds and induces a conformational change in AMPK. Liver kinase B1 (LKB1) phosphorylates AMPK and further activates AMPK [29]. We conducted *in vitro* studies to determine whether AMPK activation was required for the metformin-induced inhibition of TGF-β1-mediated fibrosis. Satriano observed a reduction in AMPK activity in the renal fibrotic tissue and showed that the induction of AMPK activity by metformin or AICAR ameliorated the severity of kidney fibrosis [30]. Several studies showed that the addition of an AMPK inhibitor (compound C) abolished the effect of metformin in decreasing fibrotic gene expression induced by TGF-β1 [9, 10, 14]. Cui showed that synthesized siRNA targeting the AMPKα1 subunit decreased the inhibitory effect of hepatocyte growth factor (HGF) on the TGF-β1-induced transition of tendon fibroblasts to myofibroblasts. However, Xiao found that metformin was directly bound to TGF-β1 and inhibited the binding of TGF-β1 to its receptor [31, 32]. In the present study, exposure to compound C abolished the inhibitory effect of metformin on TGF-β1-treated NIH/3T3 fibroblasts. These different results could be attributed to differences in the *in vitro* treatment methods and experimental conditions. Therefore, further *in vivo* studies are needed to determine whether metformin inhibits tendon adhesion by activating the AMPK signaling pathway.

In summary, recent studies have reported that metformin is an effective inhibitor of processes induced by TGF-β1 signaling. Our study provides evidence that metformin alleviated injury-induced peritendinous adhesion by inhibiting TGF-β1-induced excessive fibroblast proliferation, transition into myofibroblasts, and ECM deposition. Considering that adhesion is a common complication after tendon injury and results in various degrees of dysfunction, these findings indicate the clinical usefulness of metformin as a treatment targeting TGF-β1.

## MATERIALS AND METHODS

### Tendon injury model and treatment

Sprague-Dawley (SD) rats were purchased from the Shanghai Laboratory Animal Company (SLAC, Shanghai, China). All animal experimental procedures were approved by the Animal Care Committee of Shanghai Jiao Tong University Affiliated Sixth People's Hospital. Rats were housed in ventilated microisolator cages under a 12-h light and dark cycle with free access to food and water. For the tendon injury surgery, 8-week-old male SD rats were anesthetized by intraperitoneal injection of sodium pentobarbital at a dose of 50 mg/kg. An incision was made medially over the plantar skin, the superficial flexor tendon was resected, and then the deep flexor tendon was exposed and transected. We repaired the deep flexor tendon using a 6-0 prolene suture (Ethicon, Edinburgh, UK) and subsequently sutured the skin. After surgery, the rats were randomly assigned to the control and metformin-treated groups. The metformin-treated and control groups were orally administered 200 mg·kg^-1^·day^-1^ metformin and phosphate-buffered saline (PBS), respectively. Three weeks after tendon injury, the rats were euthanized and the tendon tissues were collected for analysis.

Macroscopic evaluation of adhesion, maximal strength, and Hyp content were performed by two independent researchers according to previously reported methods [6, 9, 13]. The severity of peritendinous adhesions, was scored as follows: grade 1, no adhesion formation; grade 2, adhesion could be separated by blunt dissection; grade 3, sharp dissection was needed to separate no more than 50% of adhesive tissues; grade 4, sharp dissection was required to separate 51–97.5% of adhesion tissues; and grade 5, sharp dissection was required to separate > 97.5% of adhesion tissues.

Peritendinous tissues were stored at -80°C or fixed in 4% paraformaldehyde and embedded in paraffin. H&E, Masson, Sirius-red, and Ki67 staining were performed according to standard procedures [9]. Histological evaluation was performed according to a previously reported system [6, 13]. Histologic adhesions were scored as follows: grade 1, no adhesions; grade 2, < 33% of the tendon surface; grade 3, 33–66% of the tendon surface; and grade 4, > 66% of the tendon surface [6, 13]. Histologic tendon healing was scored as follows: grade 1, good tendon continuity and smooth epitenon surface; grade 2, intratendinous collagen bundles exhibited good repair, but the epitenon was interrupted by adhesions; grade 3, irregularly arranged and partly broken collagen bundles; and grade 4, failed healing [6, 13].

### Cell cultures and reagents

NIH/3T3 cell lines were purchased from the Cell Bank of Type Culture Collection, Chinese Academy of Sciences (CAS, Shanghai). NIH/3T3 cells were cultured in Dulbecco's modified Eagle's medium (DMEM, Gibco) supplemented with 10% fetal bovine serum (Gibco), 100 IU/mL penicillin, and 100 ug/mL streptomycin (Gibco). Metformin, AICAR, and TGF-β1 were purchased from Sigma-Aldrich. Compound C (Selleck) was prepared in dimethyl sulfoxide (DMSO).

### Proliferation and apoptosis assays

NIH/3T3 fibroblasts were seeded at a density of 2 × 10^3^/well into 96-well plates. The cells were cultured for 24 h and then treated with TGF-β1 (2 ng/mL), metformin (5 mM), or both for 24, 48, 72, and 96 h. Cell proliferation was assessed using a CCK8 (Dojindo, Shanghai, China) after a 3-h incubation at 37°C and the absorbance was measured at a wavelength of 450 nm. EdU immunostaining was used to assess cell proliferation as previously reported. For the cell cycle analysis, cells were treated with TGF-β1, metformin, or both for 48h, followed by trypsinization and then they were fixed in 70% ethanol overnight. The fixed cells were stained with propidium iodide (PI) (50 μg/mL) for 30 minutes at room temperature [14]. For the apoptosis analysis, cells were harvested using trypsin, washed three times with PBS, and then resuspended. Then, 5 μL each of Annexin V- fluorescein isothiocyanate (FITC) and PI were added to 100 μL of the cell suspension and incubated for 30 min at room temperature. Cell apoptosis and cell cycle were analyzed using flow cytometry (Beckman Coulter, Brea, CA, USA).

### RNA isolation and real-time PCR

The mRNA expression of col1a1, col3a1, and α-SMA was analyzed using real-time PCR. Total RNA was extracted from the peritendinous tissues or cell lysates using Trizol (Invitrogen, USA) according to the manufacturer's instructions. Total RNA was reverse-transcribed into cDNA using oligo-dT primers (Promega, USA). The real-time PCR analysis was carried out using the SYBR Green Premix Ex Taq (Takara, Japan) using a Light Cycler 480 (Roche, Switzerland). Relative mRNA expression levels were calculated using comparative Ct values. Glyceraldehyde 3-phosphate dehydrogenase (GAPDH) was used as an internal reference for normalization.

### Western blotting

Cells and adhesive tendon tissues were lysed in radioimmunoprecipitation (RIPA) buffer, the lysates (20 μg) were loaded onto 10% sodium dodecyl sulfate-polyacrylamide gel electrophoresis (SDS-PAGE), and were then transferred to polyvinylidene difluoride (PVDF) membranes (Millipore, Bedford, MA, USA). The proteins were visualized using enhanced chemiluminescence (ECL) reagents (GE LifeScience, Little Chalfont, Buckinghamshire, UK) according to the manufacturer's protocol.

### IHC

For the IHC and IF of α-SMA, tendon sections and cells were incubated with the primary antibody for α-SMA in PBS solution containing 1% bovine serum albumin (BSA) overnight at 4°C. Then, the cells or peritendinous tissue sections were washed three times with PBS and incubated with secondary antibodies for 30 min at room temperature. For nuclear staining, the slides were mounted with medium with 4′, 6-diamidino-2-phenylindole (DAPI).

### Statistical analysis

All experiments were performed at least three times, and the data are described as the means ± standard error of mean (SEM). Statistical analyses were performed using the two-tailed Student's t-test. Two or more groups were compared using a one-way analysis of variance (ANOVA). All the statistical analyses were conducted using the SPSS 19.0 software (SPSS Inc., Chicago, IL, USA). A P-value < 0.05 was considered statistically significant.

## References

[1] Manske PR (1988). Flexor tendon healing. J Hand Surg Br.

[2] Xia C, Zuo J, Wang C, Wang Y (2012). Tendon Healing *in vivo*: Effect of Mannose-6-phosphate on Flexor Tendon Adhesion Formation. Orthopedics.

[3] Ishiyama N, Moro T, Ohe T, Miura T, Ishihara K, Konno T, Ohyama T, Kimura M, Kyomoto M, Saito T, Nakamura K, Kawaguchi H (2011). Reduction of Peritendinous adhesions by hydrogel containing biocompatible phospholipid polymer MPC for tendon repair. J Bone Joint Surg Am.

[4] Ishiyama N, Moro T, Ishihara K, Ohe T, Miura T, Konno T, Ohyama T, Kimura M, Kyomoto M, Nakamura K, Kawaguchi H (2010). The prevention of peritendinous adhesions by a phospholipid polymer hydrogel formed in situ by spontaneous intermolecular interactions. Biomaterials.

[5] Tang JB (2005). Clinical Outcomes Associated with Flexor Tendon Repair. Hand Clin.

[6] Jiang S, Zhao X, Chen S, Pan G, Song J, He N, Li F, Cui W, Fan C (2014). Down-regulating ERK1/2 and SMAD2/3 phosphorylation by physical barrier of celecoxib-loaded electrospun fibrous membranes prevents tendon adhesions. Biomaterials.

[7] Juneja SC, Schwarz EM, O’Keefe RJ, Awad HA (2012). Cellular and Molecular Factors in Flexor Tendon Repair and Adhesions: A Histological and Gene Expression Analysis. Connect Tissue Res.

[8] Katzel EB, Wolenski M, Loiselle AE, Basile P, Flick LM, Langstein HN, Hilton MJ, Awad HA, Hammert WC, O’Keefe RJ (2011). Impact of Smad3 Loss of Function on Scarring and Adhesion Formation during Tendon Healing. J Orthop Res.

[9] Li L, Huang W, Li K, Zhang K, Lin C, Han R, Lu C, Wang Y, Chen H, Sun F, He Y (2015). Metformin attenuates gefitinib-induced exacerbation of pulmonary fibrosis by inhibition of TGF-β signaling pathway. Oncotarget.

[10] Kim H, Moon SY, Kim JS, Baek CH, Kim M, Min JY, Lee SK (2015). Activation of AMP-activated protein kinase inhibits ER stress and renal fibrosis. Am J Physiol Renal Physiol.

[11] Xiao H, Ma X, Feng W, Fu Y, Lu Z, Xu M, Shen Q, Zhu Y, Zhang Y (2010). Metformin attenuates cardiac fibrosis by inhibiting the TGFbeta1-Smad3 signalling pathway. Cardiovasc Res.

[12] Mummidi S, Das NA, Carpenter AJ, Kandikattu H, Krenz M, Siebenlist U, Valente AJ, Chandrasekar B (2016). Metformin inhibits aldosterone-induced cardiac fibroblast activation, migration and proliferation *in vitro*, and reverses aldosterone+salt-induced cardiac fibrosis *in vivo*. J Mol Cell Cardiol.

[13] Chen S, Jiang S, Zheng W, Tu B, Liu S, Ruan H, Fan C (2017). RelA/p65 inhibition prevents tendon adhesion by modulating inflammation, cell proliferation, and apoptosis. Cell Death Dis.

[14] Cai X, Hu X, Tan X, Cheng W, Wang Q, Chen X, Guan Y, Chen C, Jing X (2015). Metformin Induced AMPK Activation, G0/G1 Phase Cell Cycle Arrest and the Inhibition of Growth of Esophageal Squamous Cell Carcinomas *in vitro* and *in vivo*. PLoS One.

[15] Cavaglieri RC, Day RT, Feliers D, Abboud HE (2015). Metformin prevents renal interstitial fibrosis in mice with unilateral ureteral obstruction. Mol Cell Endocrinol.

[16] Kita Y, Takamura T, Misu H, Ota T, Kurita S, Takeshita Y, Uno M, Matsuzawa-Nagata N, Kato K, Ando H, Fujimura A, Hayashi K, Kimura T (2012). Metformin Prevents and Reverses Inflammation in a Non-Diabetic Mouse Model of Nonalcoholic Steatohepatitis. PLoS One.

[17] Kuwahara F, Kai H, Tokuda K, Kai M, Takeshita A, Egashira K, Imaizumi T (2002). Transforming Growth Factor-beta Function Blocking Prevents Myocardial Fibrosis and Diastolic Dysfunction in Pressure-Overloaded Rats. Circulation.

[18] Mishra R, Zhu L, Eckert RL, Simonson MS (2006). TGF-beta-regulated collagen type I accumulation: role of Src-based signals. Am J Physiol Cell Physiol.

[19] Bedard K, Krause KH (2007). The NOX Family of ROS-Generating NADPH Oxidases: Physiology and Pathophysiology. Physiol Rev.

[20] Sun Y, Li F, Fan C (2016). Effect of pERK2 on extracellular matrix turnover of the fibrotic joint capsule in a post-traumatic joint contracture model. Exp Ther Med.

[21] Li F, Liu S, Ouyang Y, Fan C, Wang T, Zhang C, Zeng B, Chai Y, Wang X (2012). Effect of celecoxib on proliferation, collagen expression, ERK1/2 and SMAD2/3 phosphorylation in NIH/3T3 fibroblasts. Eur J Pharmacol.

[22] Menon SG, Goswami PC (2007). A redox cycle within the cell cycle: ring in the old with the new. Oncogene.

[23] Pardee AB (1974). A Restriction Point for Control of Normal Animal Cell Proliferation. Proc Natl Acad Sci U S A.

[24] Darby IA, Zakuan N, Billet F, Desmoulière A (2016). The myofibroblast, a key cell in normal and pathological tissue repair. Cell Mol Life Sci.

[25] Hayashida T, Decaestecker M, Schnaper HW (2003). Cross-talk between ERK MAP kinase and Smad-signaling pathways enhances TGF-β dependent responses in human mesangial cells. FASEB J.

[26] Lavoie JN, L’Allemain G, Brunet A, Müller R, Pouysségur J (1996). Cyclin D1 expression is regulated positively by the p42/p44MAPK and negatively by the p38/HOGMAPK pathway. J Biol Chem.

[27] Morgillo F, Sasso FC, Della Corte CM, Vitagliano D, D’Aiuto E, Troiani T, Martinelli E, De Vita F, Orditura M, De Palma R, Ciardiello F (2013). Synergistic Effects of Metformin Treatment in Combination with Gefitinib, a Selective EGFR Tyrosine Kinase Inhibitor, in LKB1 Wild-type NSCLC Cell Lines. Clin Cancer Res.

[28] Li L, Han R, Xiao H, Lin C, Wang Y, Liu H, Li K, Chen H, Sun F, Yang Z, Jiang J, He Y (2014). Metformin Sensitizes EGFR-TKI-Resistant Human Lung Cancer Cells *in vitro* and *in vivo* through Inhibition of IL-6 Signaling and EMT Reversal. Clin Cancer Res.

[29] Hardie DG (2008). Role of AMP-activated protein kinase in the metabolic syndrome and in heart disease. FEBS Lett.

[30] Satriano J, Sharma K, Blantz RC, Deng A (2013). Induction of AMPK activity corrects early pathophysiological alterations in the subtotal nephrectomy model of chronic kidney disease. Am J Physiol Renal Physiol.

[31] Cui Q, Fu S, Li Z (2013). Hepatocyte growth factor inhibits TGF-β1-induced myofibroblast differentiation in tendon fibroblasts: role of AMPK signaling pathway. J Physiol Sci.

[32] Xiao H, Zhang J, Xu Z, Feng Y, Zhang M, Liu J, Chen R, Shen J, Wu J, Lu Z, Fang X, Li J, Zhang Y (2016). Metformin is a novel suppressor for transforming growth factor (TGF)- β1. Sci Rep.

